# Influence of Long-Chain Amylopectin on Physicochemical and External Digestion Properties of Glutinous Rice in Zongzi

**DOI:** 10.3390/foods13060820

**Published:** 2024-03-07

**Authors:** Guangquan Li, Ling Chen, Feifei Xu, Fei Liu, Maoshen Chen, Fang Zhong

**Affiliations:** 1 State Key Laboratory of Food Science and Resources, Jiangnan University, Wuxi 214122, China; 2 Science Center for Future Foods, Jiangnan University, Wuxi 214122, China; 3 School of Food Science and Technology, Jiangnan University, Wuxi 214122, China; 4 International Joint Laboratory on Food Safety, Jiangnan University, Wuxi 214122, China

**Keywords:** glutinous rice, Zongzi, starch molecular structure, texture, coated layer, HGS

## Abstract

Zongzi, made from glutinous rice, is usually thought to stay in the stomach for a long time, causing many people to shy away. In our research, Zongzi was prepared from three indica glutinous rice samples, and three japonica glutinous rice samples were digested in vitro in a human gastric simulator (HGS). It was found that digestion performance in HGS (gastric emptying) was mainly related to the hardness and stickiness of texture properties, and surprisingly, the hardness and stickiness of Zongzi were positively correlated, which contradicts past perception. Through the extraction and analysis of the coated layer on the surface of glutinous rice grains in Zongzi, the main source of its stickiness was the entanglement between the long chains of leached amylopectin molecules. The hardness was also mainly due to the high proportion of long chains in its glutinous rice starch, which made it difficult to gelatinize. Studies suggested that stickiness gradually disappeared during digestion, while hardness had a longer impact on digestive performance. The indica glutinous rice Zongzi with a higher long-chain level showed a higher resistant-starch (RS) level and slow hydrolysis in the intestinal digestion stage. Therefore, the texture and digestibility of Zongzi can be adjusted by changing the molecular structure of glutinous rice starch.

## 1. Introduction

In China, a significant traditional food known as Zongzi is crafted from glutinous rice grains wrapped tightly in Zongzi leaf and then cooked to perfection. Therefore, the quality of Zongzi depends largely on glutinous rice, a type of rice variety with an opaque appearance, very low amylose content, and strong stickiness, and it can be divided into two kinds: japonica and indica glutinous rice [1]. At present, it is believed that glutinous rice has a more sticky texture than non-glutinous rice after cooking [2], and cooked glutinous rice stays in the digestive system for a longer time than non-glutinous rice [3], especially in the stomach, because its main physiologic functions include food mixing, emptying, and storage [4]. Still, the slower the food is emptied, the more satiety it will cause; this may be uncomfortable for people with weaker gastrointestinal digestive function.

The behavior of solid foods such as Zongzi during digestion depends on many objective factors, such as the texture of food and the structure of macromolecular nutrients (starch, protein, etc.). Hardness and stickiness are usually considered the two most important texture properties of cooked rice products [5]. The substances leached from rice during the cooking process will eventually return to the surface of the glutinous rice grains and form a coated layer after cooking, which may affect its texture [6], especially the stickiness. However, most current research focuses on the molecular mechanism underlying the texture differences of various types of rice and their differences in digestion characteristics, with glutinous rice being involved but, due to its extremely low amylose content, often used as an extreme control sample [7,8,9], which may lead to the neglect of some unique mechanisms that only belong to glutinous rice varieties. These special mechanisms may be related to factors such as the molecular structure of macromolecular nutrients in glutinous rice, such as starch and protein, which are degraded into glucose and amino acids during digestion, respectively. Pan et al. [10] compared the digestion characteristics of cooked non-glutinous and glutinous rice in an external dynamic model. Still, they did not pay attention to the effect of stickiness on digestion. Another important texture attribute is hardness; research in vitro showed that soft gel disintegrated more quickly than hard gel during digestion [11]. But, it is also unknown which plays a greater role in the digestive process (especially during gastric emptying) of Zongzi in terms of its hardness and stickiness, which is important for regulating its digestion performance. In addition, the digestion behavior of Zongzi may also be significantly different from that of conventional cooked rice due to its tightly packed cooking method; nevertheless, no pertinent research has been conducted on Zongzi as of yet.

In our research, six glutinous rice samples (including three indica glutinous rice samples and three japonica glutinous rice samples, respectively) were prepared and cooked as Zongzi to understand the influence of macro attributes, such as the texture properties of glutinous rice in Zongzi, on its digestion characteristics. We adopted an in vitro dynamic digestion model: human stomach simulator (HGS). The feature of this model is that it can simulate the disintegration and emptying of chyme in the gastric chamber in vitro, which is closer to the real situation of the human body. The physical and chemical properties of glutinous rice were analyzed, and they found a unique pattern of glutinous rice stickiness in Zongzi; afterward, a molecular-level study was conducted on the source of the stickiness difference in different Zongzi samples to establish a relationship between them and the macro texture and digestion characteristics of Zongzi, provide guidance for choosing which glutinous rice to produce food with for different functional requirements, and some ideas for glutinous rice genetic breeding research.

## 2. Materials and Methods

### 2.1. Materials

There are six milled glutinous rice samples: IGR-1, indica glutinous rice, originated from Zhuzhou city, Hunan Province, China; IGR-2, indica glutinous rice, originated from Thailand; IGR-3, indica glutinous rice, originated from Shaoyang city, Hunan Province, China; JGR-1, japonica glutinous rice, originated from Lvliang city, Shanxi Province, China; JGR-2, japonica glutinous rice, originated from Yinchuan city, Ningxia, China; and JGR-3, japonica glutinous rice, originated from the Jilin city, Jilin Province, China. Isoamylase, pepsin and pancreatin were bought from Sigma-Aldrich (St. Louis, MO, USA), and other chemicals were of analytical grade.

### 2.2. Preparation of Zongzi

The glutinous rice was weighed accurately at 70 g, and 105 g of deionized water was added before soaking for 6 h at 25 °C. After soaking, excess water was filtered with a gauze mesh, wrapped in a piece of Zongzi leaf, and tied tightly with rope to give a triangular pyramid shape-like structure; with the help of a Zongzi mold, the size and quality of Zongzi sample are basically the same. After wrapping, water was heated in the stainless-steel steamer until it boiled, and Zongzi was completely immersed in the water with 900 W of power, heated and boiled for 3 h, and stored at 25 °C. For a single batch of boiling, the water level was considered in such a way that six Zongzi samples could be immersed inside the boiling pan. 

### 2.3. Dynamic Digestion Experiment of Zongzi In Vitro

**Figure 1:**
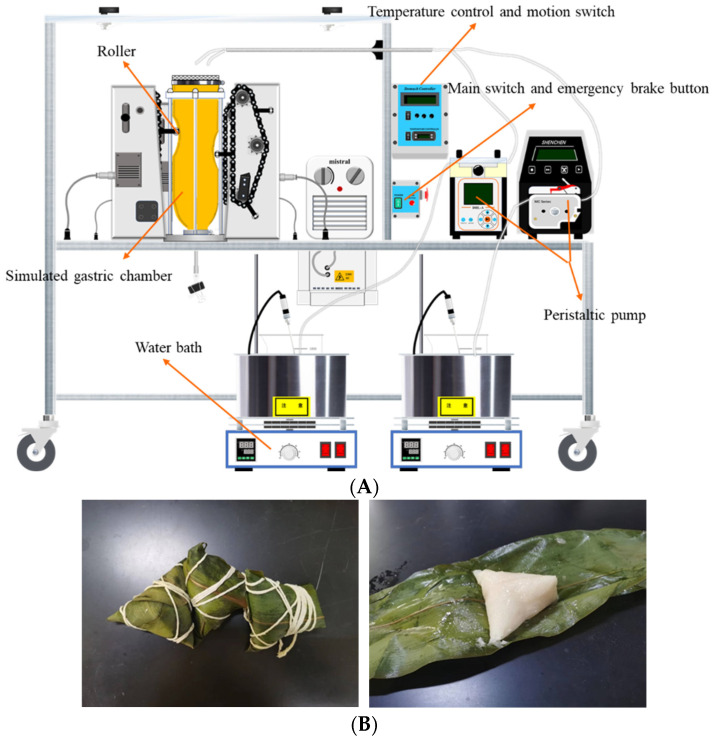
The picture of HGS and Zongzi. (**A**) HGS construction diagram. (**B**) Wrapped Zongzi and unwrapped Zongzi (cook thoroughly).

The HGS (Figure 1A) developed by Kong and Singh [12] was used to carry out the in vitro dynamic digestion simulation experiment (gastric phase) of cooked Zongzi (Figure 1B). Still, the oral and small intestine digestion stages were in vitro static digestion simulation experiments. Refer to Minekus et al. [13] to prepare simulated salivary fluid (SSF), simulated gastric fluid (SGF), and simulated intestinal fluid (SIF).

In vitro oral digestion stage: one hundred grams of cooked Zongzi (unwrapped) was crushed and reacted with 20 mL of SSF containing α-amylase (pH = 7.00) at 37 °C for 2 min. In vitro gastric digestion stage: 10 mL of SGF (pH = 3.00) was added to HGS to simulate the fasting state of the human body with HGS internal environment temperature at 37 °C. Two peristaltic pumps delivered SGF-1 and SGF-2 at different flow rates and were kept warm in a 37 °C water bath. SGF-1 was a concentrated simulated gastric juice without enzymes, and SGF-2 was an aqueous gastric protease solution containing CaCl_2_. The two are finally mixed in HGS to produce the correct electrolyte ratio. After the oral digestion stage, the oral digestive mixture was transferred to the HGS gastric chamber, and the motion switch was turned on. The digestion time was adjusted to 120 min. Under the continuous compression of the electric motor driving the surrounding rollers, the gastric chamber contracted at a frequency of 3 times per min to simulate the contraction of the human stomach. Due to the presence of a mesh bag in the simulated gastric chamber, which serves as a sieving effect of the pylorus, only 1~2 mm of food particles are allowed to pass through and empty. During this period, 20 mL of chyme was collected below the gastric bag for further analysis at 0, 20, 40, 60, 80, 100, and 120 min, respectively. In vitro small intestine digestion stage: the method was referred to by Iqbal et al. [14].

#### 2.3.1. Chyme Emptying Characteristics during the Gastric Digestion Phase

The method was referred to by Guo et al. [11]. The gastric emptying characteristics were expressed as the percentage of chyme left in the simulated gastric chamber at different times, which was the gastric retention rate. The specific operation was to place the collected chyme at different times in an oven at 105 °C for 24 h dry to constant weight, then calculate the cumulative dry weight of chyme emptied from HGS. The calculation formula for the gastric retention rate is as follows:

(1) $$y(t)=\frac{\left(W_{0}-W_{t}\right)}{W_{0}}\times 100\%$$

where y(t) is the percentage content remaining in the stomach at time t; W_0_ is the initial dry weight; and W_t_ is the dry weight of the accumulated emptied chyme at time t.

Subsequently, the optimized Elashoff model [15], through the custom model fitting function of software 1stOpt 1.5, obtained the value of k and β:

(2) $$y(t)=1-{\left(1-e^{-kt}\right)}^{\beta }$$

where k is the gastric emptying rate (min^−1^), and β wi the extrapolated y-intercept of the end of the curve.

The calculated gastric emptying lag time T_lag_ and gastric half-emptying time t_1/2_ is as follows:

(3) $$T_{\mathrm{lag}}=\frac{ln\beta }{k}$$

(4) $$t_{1/2}=-\frac{1}{k}ln(1-0.5^{\frac{1}{\beta }})$$

#### 2.3.2. Changes in the Size of Emptying Chyme during the Gastric Digestion Phase

The method was referred to by Nadia J et al. [16]. The collected chyme was analyzed by a Malvern laser particle-size analyzer at different times, with water as the dispersion medium.

#### 2.3.3. Changes in Viscosity of Emptying Chyme during Gastric Digestion Phase

The collected chyme was placed at different time points on a rheometer Peltier with a 40 mm plate clamp and a Flow Peak Hold test mode. The test temperature was 37 °C, with a 120 s duration and a 30 s^−1^ shear rate.

#### 2.3.4. Changes in pH Value of Emptying Chyme during Gastric Digestion Phase

The collected chyme at different times was immediately measured with a pH meter and then recorded.

#### 2.3.5. Protein Digestion during Gastric Digestion Phase

The protein components in food are mainly digested during the gastric stage. The study primarily used the ninhydrin colorimetric method proposed by Jarunglumlert et al. [17] to determine the release of amino acids to characterize protein digestion during this process.

#### 2.3.6. Glucose Release during In Vitro Small Intestine Digestion Phase

The GOPOD method was used to determine the glucose content produced during starch digestion in the small intestine phase, and the levels of RDS (rapidly digesting starch), SDS (slowly digesting starch), and RS (resistant starch) were calculated, refer to Englyst [18], with the following equations:

(5) $$\mathrm{RDS}(\%)=\frac{(G_{20}-FG)\times 0.9\times 100}{TS}$$

(6) $$\mathrm{SDS}(\%)=\frac{(G_{120}-G_{20})\times 0.9\times 100}{TS}$$

RS (%) = 100% − RDS (%) − SDS (%)(7)

Here, 0.9 is the conversion coefficient; TS is the total starch content; FG is the free glucose content; G_20_ is the glucose content during digestion for 20 min; and G_120_ is the glucose content at 120 min of digestion.

For the determination of the starch hydrolysis curve, the starch hydrolysis % was calculated at 0, 20, 40, 60, 80, 100, and 120 min, respectively, and the starch hydrolysis curve follows the first order kinetic equation [19], which is as follows:

C_t_ = C_∞_ (1 − e^−kt^) (8)

where C_t_ is the starch hydrolysis % at time t; C_∞_ is the starch hydrolysis % at the equilibrium of the digestion reaction; and k is the first-order kinetic constant. C_∞_ and k values were obtained using the OriginPro 8.5 nonlinear fitting function.

### 2.4. Determination of Texture Properties of Zongzi

The cooked Zongzi was cut into a rectangle with a thickness of 1 cm, a length of 3 cm, a width of 2 cm, and a weight of 8 g. It was placed on the texture analyzer (TA. XTplus100, Stable Micro Systems, Surrey, UK) test bench in TPA mode, with a probe of P 0.5 and a force-sensing element of 5 kg. The test conditions were as follows: the pre-test speed was 1 mm s^−1^, the test speed was 0.8 mm s^−1^, the post-test speed was 0.6 mm s^−1^, the deformation degree was 60%, the compression time was 5 s, and the triggering force was 5 g.

### 2.5. Extraction of a Coated Layer of Cooked Zongzi

The coated layer was extracted from the surface of the rice of cooked Zongzi, according to the method proposed by Tao et al. [20] with slight modifications.

### 2.6. The Analysis of the Coated Layer of Cooked Zongzi

#### 2.6.1. Components of the Coated Layer of Cooked Zongzi

After freeze-drying treatment, the weight of the coated layer was the amount of leached substance during the cooking process; through the Coomassie blue staining method, the protein content of the coated layer was obtained; the total starch content of the coated layer was determined by referring to the method proposed by Lin et al. [21] with slight modifications.

#### 2.6.2. Starch Molecular Structure of the Coated Layer of Cooked Zongzi

##### Molecular Weight of Starch in Coated Layer

For sample preparation, the sample was accurately weighed at 50 mg and placed in a 15 mL covered centrifuge tube equipped with a magnetic rotor. A volume of 10 mL of DMSO solution containing 0.5% LiBr (*w*/*w*) was added, heated in a boiling water bath, stirred for 30 min until dissolved, and stirred overnight at 50 °C. A volume of 1 mL of sample solution was taken and centrifuged for 10 min at 10,000 rpm, and the upper liquid was added to the organic filter membrane with a pore size of 0.22 μm to be prepared for sample injection measurement.

HPSEC-MALLS-RI (Wyatt Technologies Corporation, Goleta, CA, USA) chromatographic conditions were as follows: the mobile phase was a DMSO solution containing 0.5% LiBr (*w*/*w*) (filtered by 0.22 μm organic filter membrane, ultrasonic degassing), with a flow rate of 0.5 mL/min, dn/dc of 0.07, and column temperature set at 45 °C. The average molecular weight (Mw) was analyzed using the ASTRA 5.3.4 software. The fitting model used was the second-order berry model.

##### Chain Length Distribution of Starch in the Coated Layer

For the sample preparation method, the sample was accurately weighed at 50 mg and placed in a 15 mL covered centrifuge tube equipped with a magnetic rotor. Ten milliliters of sodium acetate buffer (0.05 mol/L, pH = 3.5) was added, heated, and stirred in a boiling water bath for 30 min to gelatinize and disperse thoroughly. After cooling, 2 mL of starch solution was taken and placed in a 5 mL centrifuge tube equipped with a rotor, then 500 μL of isoamylase solution was added to it and it was placed in a 40 °C water bath, continuously stirred for hydrolysis and debranching, for 24 h. After debranching, it was heated and stirred in a boiling water bath for 30 min to terminate the reaction. The rotor was removed and centrifuged at 10,000 rpm for 10 min. Then, 0.1 mL of the supernatant was diluted with 4.9 mL of sterilized ultrapure water and vortexed, passing through a 0.22 μm water filter membrane, and injected into HPAEC-PAD (DIONEX ICS-5000+SP-5, Thermo Fisher Scientific, Waltham, MA, USA) for testing.

After the test was completed, according to the Hanashiro I et al. [22] method, the percentages of A chain, B_1_ chain, B_2_ chain, and B_3_ chain were calculated separately.

The average chain length (ACL) was calculated using the method of Koch et al. [23]:

(9) $$\bar{DP}=\frac{\sum A_{i}\times {DP}_{i}}{\sum A_{i}}$$

where i is the serial number of the peak, A is the peak area, and DP is the degree of polymerization.

### 2.7. Components of Glutinous Rice

The determination method for the apparent amylose content in glutinous rice referred to the Chinese national standard GB/T 15683-2008 [24]; the protein content of glutinous rice was determined by referring to the Kjeldahl method in the GB 5009.5-2016 [25]; the lipid content of glutinous rice was determined by referring to the Soxhlet extraction method in the GB 5009.6-2016 [26]; the ash content of glutinous rice was determined by referring to the first method in the GB 5009.4-2016 [27]; the total starch content of glutinous rice was determined by referring to the method proposed by Lin et al. [21] with slight modifications; and the moisture content of glutinous rice was determined by referring to the first method in the GB 5009.3-2016 [28].

### 2.8. Extraction of Glutinous Rice Starch

Preparation of glutinous rice starch using alkaline leaching method was as follows: 30 g glutinous rice flour was taken, and a 0.4% (*w*/*v*) NaOH solution with a solid-liquid ratio of 1:5 (g/mL) was added; the extraction time was 24 h, and continuous stirring was carried out during the soaking process. After soaking, the supernatant was centrifuged at 10,000 rpm for 10 min, the precipitate was separated, and the yellow soft substance on the surface was scraped off. The precipitate was then remade into a suspension using deionized water. The HCl solution with a concentration of 1 mol/L was adjusted to medium and centrifuged again. The supernatant was discarded, and the precipitate was washed with deionized water multiple times. The residue was dried in an oven at 40 °C for 24 h and crushed before sieving to obtain glutinous rice starch. 

### 2.9. Determination of the Molecular Structure of Glutinous Rice Starch

The method was the same as used in Section 2.6.2.

### 2.10. Differential Scanning Calorimeter (DSC) Analysis

Accurately weighed 2 mg of starch sample was placed in a pan, where 6 μL of deionized water was added, sealed, and kept in a refrigerator at 4 °C overnight. DSC (Netzsch, Bayern, Germany) testing conditions were as follows: the temperature range was 20~120 °C, and the heating rate was 10 °C/min. After the test, software analysis obtained the onset gelatinization temperature T_o_, peak gelatinization temperature T_p_, conclusion gelatinization temperature T_c_, and gelatinization enthalpy ΔH.

### 2.11. Statistical Analysis

The above data results were analyzed using software SPSS 22, one-way ANOVA, and Duncan multiple comparisons. If the differences between sample data are significant (*p* < 0.05), different letters are marked, and the OriginPro 8.5 software is used for plotting.

## 3. Results and Discussion

### 3.1. Analysis of Digestion Characteristics of Zongzi In Vitro

#### 3.1.1. Analysis of Gastric Emptying Characteristics of Zongzi In Vitro

**Figure 2:**
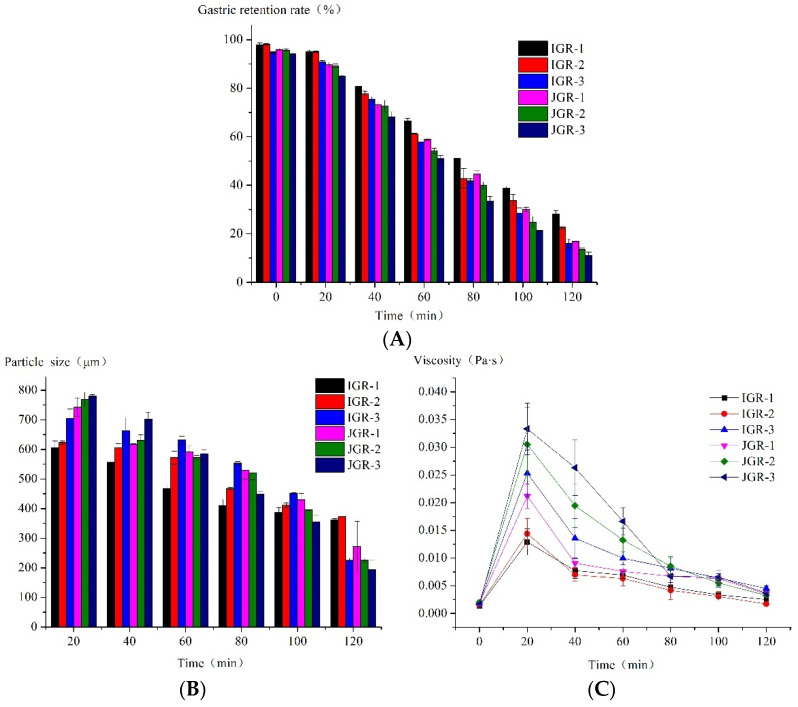
The properties of gastric emptying chyme during HGS digestion. (**A**) Changes in gastric retention rate during the HGS digestion process. (**B**) Changes in particle size during the HGS digestion process. (**C**) Changes in the viscosity of emptying chyme during the HGS digestion process.

Gastric emptying is a complex and important phenomenon that transfers food digesta from the stomach to the duodenum after digestion. As shown in Table 1, the factor β determines the shape of the gastric emptying curve. The β values of six Zongzi samples were all greater than 1, indicating a lag period in the initial emptying stage. Figure 2A also showed the phenomenon of lag emptying; it is generally believed that the emptying lag time T_lag_ is defined as the time required to grind and crush food to a particle size that can pass through the pylorus. The final gastric retention rate of different Zongzi samples at the end of digestion was approximately 11.06% to 28.11%. Ranganathan et al. [29] used the dynamic digestion model ARK^®^ in their research, and they found that only about 15% of the solid components of the rice sample remained in the machine at the end of 120 min of digestion. The amount of chyme remaining in HGS decreased gradually with digestion progression. The T_lag_ and t_1/2_ of indica glutinous rice Zongzi were significantly higher than that of japonica glutinous rice Zongzi, indicating that indica glutinous rice Zongzi stayed in the stomach chamber longer and was harder to empty. The japonica glutinous rice Zongzi had undergone more severe disintegration in this process, and the final gastric retention rates of IGR-1 and IGR-2 Zongzi were significantly higher than those of other samples, but JGR-3 Zongzi had the lowest. These phenomena during the digestion process may be related to factors such as their texture properties.

In Figure 2B, the particle size data at 0 min were not collected because the amount of chyme emptied at 0 min was low and could not meet the test required for the laser particle size analyzer. It could be seen that the particle size of the chyme in all samples decreased over time, indicating that the samples undergo disintegration under the action of mechanical force, enzymes, and acids. The alterations in the particle size of chyme in IGR-1 and IGR-2 Zongzi during digestion were comparatively mild to other samples, indicating that its food structure was relatively stable; however, JGR-2 and JGR-3 Zongzi were especially prone to structural damage, large particle-sized fragments were generated from the initial stage, and their chyme particle size continued to rapidly decrease as digestion progressed, consistent with the pattern of gastric emptying characteristics.

In Figure 2C, at the time of 0 min, when the oral digestive mixture was first added to HGS, the food structure was relatively intact, with large particles and minimal emptying, resulting in a very low chyme viscosity, and thus explains why the samples collected at 0 min did not satisfy the test criteria of the laser particle size analyzer. It was seen that the viscosity of all chyme decreased with time, consistent with the decreasing trend in chyme particle size over time mentioned above, which indicates that food undergoes severe disintegration. And, except for IGR-1 and IGR-2 Zongzi, the viscosity of chyme in other samples changed greatly over time, especially JGR-3 Zongzi; at 20 min, its chyme viscosity was highest because a large number of food fragments with larger particle sizes were produced, but due to the fast emptying speed, the viscosity of JGR-3 chyme also decreased rapidly over time; this was confirmed by the phenomenon pattern of gastric emptying characteristics.

#### 3.1.2. Analysis of Protein Digestion of Zongzi In Vitro

**Figure 3:**
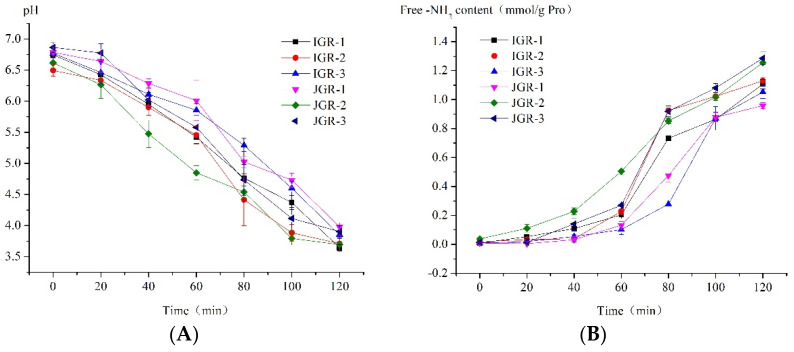
Changes in protein in Zongzi during HGS digestion. (**A**) Changes in pH value during the HGS digestion process. (**B**) Changes in amino acid levels are released by protein hydrolysis during the HGS digestion process.

pH value plays an extremely important role in digestion, as it affects the structure of food intake and enzyme activity. Figure 3A shows the pH dynamic change in HGS due to the dilution and buffering effect of the sample, as well as the consumption of hydrochloric acid. The environmental pH value almost reached neutral when food first entered the HGS; later, over time, due to the continuous secretion of gastric juice and emptying of chyme, the pH value gradually decreased to near the pH value of the fasting state, similar to the actual situation in the body. Different samples have different buffering abilities due to factors such as differences in grain composition. JGR-1 Zongzi showed strong buffering ability among samples; however, JGR-2 Zongzi exhibited weak buffering ability.

The stomach is the main organ for protein digestion. Figure 3B shows the trend in the amount of amino acids released by protein hydrolysis during the dynamic digestion process in the stomach stage. It could be observed that the level of free amino acids in the system increased with digestion, and before 60 min, the degree of protein hydrolysis was not high, but after 60 min, it rapidly increased; it was speculated that at some point between 60 and 80 min, the working pH value of pepsin was reached (pepsin began to become active below pH = 4.5–5 [30]), and the protein rapidly hydrolyzed. The weak buffering ability of JGR-2 Zongzi led to a rapid decrease in pH, and pepsin began to hydrolyze earlier; in contrast, JGR-1 Zongzi had a strong buffering ability and caused slow protein hydrolysis. JGR-3 Zongzi had the highest degree of protein hydrolysis at the end of digestion in the gastric stage; this might be related to its greater degree of disintegration, as the destruction of food structure was conducive to the action of pepsin. 

#### 3.1.3. Starch Hydrolysis Rate during Static Small Intestine Digestion

The small intestine is the main part of starch digestion, and different starch nutritional fragments have various physiological effects; the intake of RDS in food will lead to a rapid rise in the blood sugar level of the body, and it is often associated with negative effects such as diabetes. SDS is between RDS and RS, with a slow digestion rate and the ability to continuously supply glucose to the body and maintain blood glucose homeostasis; therefore, from a nutritional perspective, SDS is the ideal starch type [31]. RS is not digested in the small intestine but enters the large intestine for fermentation, it can produce a large amount of short-chain fatty acids (such as butyrate, acetate, etc.) through fermentation by intestinal microorganisms, which is beneficial for human intestinal health [32]. From the Table 2 data, it was seen that except for IGR-1 and JGR-3, there was not much difference in SDS levels among samples. Still, the RS levels of all samples in this study ranged from 13.07 to 25.64%, comparable to the results reported by Wang et al. [33], and the overall RS level of indica glutinous rice Zongzi was higher than that of japonica glutinous rice Zongzi. The overall RDS level, k, and C_∞_ of japonica glutinous rice Zongzi were higher than that of indica glutinous rice Zongzi. Therefore, it is believed that japonica glutinous rice was more likely to cause an increase in blood sugar compared to indica glutinous rice in Zongzi.

### 3.2. Texture Attribute Analysis of Zongzi

The texture attribute of Zongzi was achieved through a texture analyzer. Table 3 shows that except for cohesiveness and chewiness, there were significant differences among samples, and the hardness and stickiness of indica glutinous rice Zongzi were roughly higher than those of japonica glutinous rice Zongzi.

Combined with the digestion phenomenon of different Zongzi samples during HGS digestion, indica glutinous rice Zongzi, particularly IGR-1 and IGR-2, exhibited greater resistance to emptying and disintegration, attributed to their high hardness and stickiness. The firm structure resists the mechanical forces in the stomach, while strong stickiness between glutinous rice grains has a similar resistant effect. Although rice grains absorb digestive juices and soften, continuous secretion of digestive juices eventually damages the coated layer, diminishing stickiness. The impact of stickiness persists until its complete disappearance. 

As for which property of hardness and stickiness played a stronger and more lasting role in this process, a judgment could be made through IGR-3 and JGR-1: the hardness and final gastric retention rate of JGR-1 were greater than that of IGR-3, but the stickiness and T_lag_ of JGR-1 were smaller than that of IGR-3. Therefore, stickiness mainly worked in the early stages of digestion, leading to delayed emptying, but was followed by a damaged coated layer and decreased stickiness. However, the hardness maintains its effect for longer, resulting in more samples remaining in the gastric chamber.

### 3.3. Mechanism of Zongzi Stickiness Differences

In previous studies, it was generally believed that there was a negative correlation between the hardness and stickiness of cooked rice and the stickiness source of the short chain of amylopectin, but most focused on non-glutinous rice [5]. In our research, we found that the hardness and stickiness of glutinous rice Zongzi were positively correlated; after cooking, indica glutinous rice generally exhibited stronger hardness and stickiness than japonica glutinous rice. Surprisingly deviating from prior patterns, our focus was on the coated layer, a key factor in rice stickiness.

#### 3.3.1. Composition and Starch Molecular Structure of Coated Layer from Zongzi

As indicated in Table 4, the substance leached during cooking formed a coated layer, primarily composed of starch. Additionally, Table 5 revealed that the indica glutinous rice-coated layer exhibited higher levels of long B_3_ chain and ACL in starch molecules than the japonica glutinous rice-coated layer. Conversely, the short A-chain level in the japonica glutinous rice-coated layer surpassed that of the indica glutinous rice-coated layer.

#### 3.3.2. Correlation Analysis of Zongzi Stickiness and Coated-Layer Physicochemical Properties

The correlation between the stickiness of Zongzi, the coated layer component, and the molecular structure of starch in the coated layer is shown in Table 6 and Table 7. There was a significant positive correlation between stickiness and the long B_3_ chain of the starch molecule in the leachate (r = 0.83*, *p* < 0.05). It was found that the amount of leachate and leached starch had no significant effect on stickiness. However, earlier research suggested that the more leached substances during rice cooking, the greater the stickiness of the cooked rice [34], but the study samples were non-glutinous rice. In another research, Li et al. thought that the stickiness difference of rice was mainly from the amount of leached substances rather than starch molecular structure [35]. Later, it was claimed that high-amylose rice contains amylose molecules that inhibit swelling and starch leaching, resulting in a hard and non-sticky texture [36]. One of the main differences between glutinous rice and non-glutinous rice was the amylose content, which might lead to the fact that the amount of leached substances and the amount of leached starch in this research were not the important factors for the stickiness difference of cooked glutinous rice. Due to genetic characteristics, glutinous rice is almost entirely composed of amylopectin, so the inhibitory effect of amylose can be ignored. The negligible variation in the quantity of leached substances and starch among distinct samples further supports this observation. The fine structure of the leached starch molecules had a significant impact on the stickiness of cooked glutinous rice in Zongzi, especially with long B_3_ chains; long chains between the leached starch molecules tended to entangle with each other, leading to an increase in stickiness. In addition, there was a positive correlation between the M_w_ and stickiness, which could be due to the fact that larger molecules have more opportunities to make contact with each other. 

### 3.4. Components of Glutinous Rice

Table 8 displays the components of six raw glutinous rice samples, computed on a dry basis, excluding moisture content. The results showed that starch was the main component of milled glutinous rice, whereas protein and other substances were minor components. The apparent amylose content of six samples ranged from 0.65% to 5.37%. Due to the absence of granule-bound starch synthase 1 (GBSS 1) (responsible for the biosynthesis of amylose) in glutinous rice, there was almost none or a very small amount of amylose [37], and the apparent amylose content was composed of real amylose and long chains of amylopectin. Therefore, it was inferred that the apparent amylose content of glutinous rice was mainly contributed by the long chains of amylopectin [38]. These data showed that the apparent amylose content of indica glutinous rice was significantly higher (*p* < 0.05) than that of japonica glutinous rice.

Regarding the impact of protein content in samples on digestion performance, JGR-1 showed strong buffering ability during HGS digestion, possibly due to high protein content. Research has shown that foods with higher protein content have higher buffering capacity [39]. Conversely, JGR-2 exhibited weak buffering ability due to low protein content. Surprisingly, IGR-3 also showed powerful buffering ability. However, its protein content was low, possibly associated with amino acid composition. Previous research investigated that the higher the level of acidic amino acids in the protein, the stronger its buffering ability [40]. The weak correlation between the protein hydrolysis curve and gastric emptying pattern can be attributed to the low protein content in rice, which had an insignificant effect on gastric emptying.

### 3.5. Molecular Structure of Glutinous Rice Starch

After analyzing the basic components of raw glutinous rice mentioned above, it was found that starch was the main component of glutinous rice grains. Therefore, the molecular structure of starch might play an important role in the macroscopic properties of glutinous rice. The results of obtaining the fine structure of the glutinous rice starch are shown in Table 9. The proportion of short A chain in japonica glutinous rice starch was roughly higher than in indica glutinous rice starch. The proportion of long B_3_ chain and ACL in japonica glutinous rice starch was approximately lower than in indica glutinous rice starch. The data proportion of the long B_3_ chain and ACL were consistent with the apparent amylose content of the sample, which proved the contribution of the long chain of amylopectin to the apparent amylose content to some extent.

When comparing natural glutinous rice starch and the coated layer starch molecule, the molecular weight of the coated layer starch molecule was much smaller than that of natural glutinous rice starch, and coated layer starch molecules have more short A chain, fewer B_1_ chain, B_2_ chain, and shorter ACL. It was considered that glutinous rice was mainly composed of amylopectin; the leached starch might consist of smaller size amylopectin molecules, with an extremely little amount of protein leaching, because during cooking, smaller starch molecules easily escaped from the starch frame when starch granules were heated and destroyed. However, large amylopectin molecules might tend to entangle between chains to maintain stable granule structures [41].

In addition, the starch molecule in the coated layer also exhibited a pattern similar to the chain length distribution in natural glutinous rice starch. Compared with japonica glutinous rice, natural and coated layer starch molecules of indica glutinous rice had higher long B_3_ chain and ACL levels, but lower short A-chain levels. From this, it could be ascribed that the cooking process might not modify the structure of the starch chain length.

### 3.6. Thermal Properties of Glutinous Rice Starch Samples

Through the DSC analysis, with the T_o_, T_p_, T_c,_ and ΔH shown in Table 10, the T_o_, T_p_, T_c,_ and ΔH in japonica glutinous rice starch was found to be approximately lower than that in indica glutinous rice starch. The result indicated that indica glutinous rice starch was more difficult to gelatinize than japonica glutinous rice starch, which is consistent with the chain length distribution data mentioned above. As a result, long chains contribute to starch granule structure stability, while short A-chains can lead to defects in crystalline structure [42]. Moreover, long chains might be more likely to interact with components in grain, such as proteins [43]; its structure is relatively stable so that indica glutinous rice Zongzi may show greater hardness after cooking. 

According to the above discussion, the hardness and stickiness of Zongzi were controlled by the long chains of starch molecules in glutinous rice and coated layer, respectively. The higher RS level of indica glutinous rice Zongzi during the in vitro small intestine digestion stage might also be related to the long-chain level of their starch molecule [44].

## 4. Conclusions

**Figure 4:**
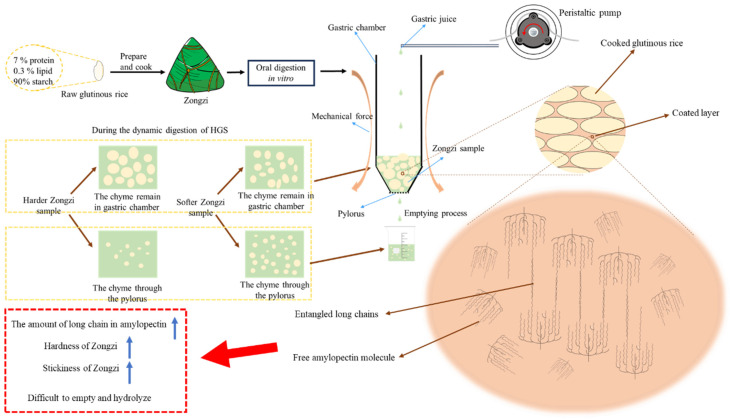
Mechanism diagram of dynamic digestion characteristics of glutinous rice Zongzi.

The research (Figure 4) performed an in vitro dynamic simulation digestion experiment on Zongzi derived from six glutinous rice samples. The investigation aimed to explore the relationship between observed phenomena and the physicochemical qualities of glutinous rice. The results indicated that indica glutinous rice exhibited a higher level of long-chain starch molecules than japonica glutinous rice. This difference made the gelatinization process more challenging for indica glutinous rice, ultimately resulting in an increased hardness in the texture of Zongzi. In addition, when the long-chain level of starch molecules leached from cooked glutinous rice was higher, and the molecular chains became entangled, the resultant Zongzi exhibited increased stickiness. The chain length distribution of natural starch was consistent with that of starch molecules in the coated layer, contributing to the distinct characteristics of robust hardness and stickiness in the indica glutinous rice Zongzi. This particular feature caused indica glutinous rice Zongzi to be more resistant during in vitro dynamic gastric digestion, leading to a slower emptying process. As a result, indica glutinous rice Zongzi, with a longer chain, showed increased resistant starch (RS) levels during the intestinal digestion stage and experienced more gradual hydrolysis.

Therefore, possible deduction revealed the influencing factor of amylopectin chains on the physicochemical and digesting qualities of glutinous rice in Zongzi. The texture and digestibility of Zongzi can be adjusted to meet cultural, health, and consumer preferences by changing the molecular structure of glutinous rice starch, presenting a unique opportunity for the industry to carve out a specialized niche in the market.

## Figures and Tables

**Table 1:** Gastric emptying parameters by fitting the optimized Elashoff model.

Sample	k	β	T_lag_ (min)	t_1/2_ (min)
IGR-1	0.02 ± 0.00 ^c^	2.38 ± 0.09 ^ab^	52.17 ± 0.23 ^a^	83.33 ± 1.61 ^a^
IGR-2	0.02 ± 0.00 ^b^	2.45 ± 0.03 ^ab^	47.26 ± 0.74 ^b^	73.80 ± 0.64 ^b^
IGR-3	0.02 ± 0.00 ^ab^	2.51 ± 0.31 ^a^	43.81 ± 2.60 ^bc^	68.18 ± 0.35 ^c^
JGR-1	0.02 ± 0.00 ^b^	2.15 ± 0.09 ^ab^	40.44 ± 2.32 ^c^	68.06 ± 2.10 ^c^
JGR-2	0.02 ± 0.00 ^a^	2.37 ± 0.15 ^ab^	40.10 ± 1.68 ^c^	63.79 ± 0.46 ^d^
JGR-3	0.02 ± 0.00 ^a^	2.09 ± 0.12 ^b^	34.23 ± 1.39 ^d^	58.83 ± 0.23 ^e^

Values are shown by Mean ± SD and values, and different letters within a column indicate significant differences between mean values (*p* < 0.05).

**Table 2:** In vitro small intestine digestion stage data.

Sample	RDS (%)	SDS (%)	RS (%)	C_∞_ (%)	k (min^−1^)
IGR-1	47.18 ± 0.27 ^e^	27.18 ± 0.53 ^a^	25.64 ± 0.27 ^a^	70.27 ± 0.74 ^e^	0.06 ± 0.00 ^d^
IGR-2	56.93 ± 1.19 ^d^	21.57 ± 1.33 ^b^	21.50 ± 0.13 ^b^	71.88 ± 0.21 ^e^	0.08 ± 0.00 ^c^
IGR-3	62.00 ± 1.38 ^b^	20.91 ± 1.04 ^b^	17.09 ± 0.35 ^c^	79.82 ± 1.82 ^c^	0.10 ± 0.00 ^b^
JGR-1	59.30 ± 1.07 ^c^	23.65 ± 0.24 ^b^	17.04 ± 1.30 ^c^	77.31 ± 0.33 ^d^	0.10 ± 0.00 ^b^
JGR-2	64.28 ± 0.35 ^b^	22.66 ± 2.79 ^b^	13.07 ± 2.44 ^d^	83.36 ± 0.28 ^b^	0.10 ± 0.00 ^b^
JGR-3	72.23 ± 0.74 ^a^	11.35 ± 0.42 ^c^	16.41 ± 0.32 ^c^	92.34 ± 0.33 ^a^	0.15 ± 0.01 ^a^

Values are shown by Mean ± SD and values, and different letters within a column indicate significant differences between mean values (*p* < 0.05).

**Table 3:** Texture attribute data of Zongzi.

Sample	Harness (g)	Stickiness (g·s)	Springiness	Cohesiveness	Chewiness
IGR-1	562.21 ± 35.94 ^a^	464.30 ± 38.00 ^ab^	0.75 ± 0.03 ^a^	0.44 ± 0.03 ^a^	177.94 ± 12.99 ^a^
IGR-2	533.06 ± 27.31 ^ab^	501.68 ± 9.10 ^a^	0.75 ± 0.07 ^a^	0.47 ± 0.01 ^a^	172.00 ± 11.78 ^a^
IGR-3	460.67 ± 15.77 ^bc^	423.98 ± 32.26 ^abc^	0.76 ± 0.01 ^a^	0.47 ± 0.09 ^a^	166.32 ± 26.19 ^a^
JGR-1	523.95 ± 53.54 ^abc^	342.92 ± 3.79 ^c^	0.65 ± 0.01 ^bc^	0.45 ± 0.03 ^a^	148.33 ± 11.63 ^a^
JGR-2	438.49 ± 49.67 ^cd^	395.52 ± 92.91 ^abc^	0.72 ± 0.00 ^ab^	0.45 ± 0.00 ^a^	156.03 ± 24.80 ^a^
JGR-3	356.76 ± 2.04 ^d^	376.78 ± 4.97 ^bc^	0.59 ± 0.01 ^c^	0.42 ± 0.09 ^a^	87.78 ± 19.49 ^b^

Values are shown by Mean ± SD and values, and different letters within a column indicate significant differences between mean values (*p* < 0.05).

**Table 4:** Composition and starch molecular weight of coated layers.

Sample	Coated Layer Weight (mg)	Total Starch Content (%)	Protein Content (%)	M_w_ (×10^7^ g/mol)
IGR-1	408.70 ± 9.90 ^a^	81.59 ± 3.12 ^a^	0.50 ± 0.06 ^ab^	1.06
IGR-2	411.40 ± 4.67 ^a^	82.25 ± 0.58 ^a^	0.34 ± 0.14 ^bc^	1.32
IGR-3	423.60 ± 13.86 ^a^	83.60 ± 0.47 ^a^	0.68 ± 0.08 ^a^	1.09
JGR-1	413.00 ± 0.57 ^a^	82.98 ± 2.63 ^a^	0.19 ± 0.06 ^c^	1.12
JGR-2	415.50 ± 4.81 ^a^	78.97 ± 4.90 ^a^	0.34 ± 0.02 ^bc^	1.10
JGR-3	413.75 ± 5.02 ^a^	83.61 ± 1.82 ^a^	0.56 ± 0.07 ^a^	1.19

Values are shown by Mean ± SD and values, and different letters within a column indicate significant differences between mean values (*p* < 0.05).

**Table 5:** Chain length distribution of starch in coated layer.

Sample	A _(DP 6–12)_	B_1 (DP 13–24)_	B_2 (DP 25–36)_	B_3 (DP≥37)_	ACL
IGR-1	30.97	45.99	11.46	11.59	19.72
IGR-2	33.43	43.12	11.09	12.36	19.78
IGR-3	33.11	44.81	10.60	11.48	19.30
JGR-1	31.64	46.07	11.01	11.28	19.52
JGR-2	33.64	44.25	10.74	11.38	19.30
JGR-3	35.60	43.43	10.19	10.78	18.84

**Table 6:** Correlation coefficient of Zongzi stickiness and composition of the coated layer.

	Coated Layer Weight	Total Starch Content	Protein Content
Stickiness	−0.24	−0.12	0.23

**Table 7:** Correlation coefficient of Zongzi stickiness and starch molecular structure of the coated layer.

	M_w_	A	B_1_	B_2_	B_3_	ACL
Stickiness	0.41	−0.16	−0.31	0.5	0.83 *	0.62

* indicates significant difference with stickiness (*p* < 0.05).

**Table 8:** Components of raw glutinous rice samples.

Sample	Apparent Amylose Content (%)	Protein Content (%)	Water Content (%)	Lipid Content (%)	Ash Content (%)	Total Starch Content (%)
IGR-1	5.37 ± 0.10 ^a^	6.44 ± 0.15 ^e^	12.37 ± 0.04 ^c^	0.15 ± 0.01 ^c^	0.15 ± 0.01 ^bcd^	90.07 ± 0.04 ^a^
IGR-2	2.84 ± 0.24 ^b^	7.11 ± 0.00 ^c^	10.99 ± 0.08 ^d^	0.18 ± 0.00 ^c^	0.20 ± 0.02 ^ab^	89.18 ± 0.06 ^d^
IGR-3	2.34 ± 0.02 ^c^	6.83 ± 0.03 ^d^	12.53 ± 0.01 ^c^	0.42 ± 0.01 ^a^	0.14 ± 0.02 ^cd^	87.57 ± 0.02 ^f^
JGR-1	1.72 ± 0.12 ^d^	7.86 ± 0.15 ^a^	13.08 ± 0.08 ^b^	0.30 ± 0.02 ^b^	0.11 ± 0.04 ^d^	89.55 ± 0.03 ^c^
JGR-2	1.12 ± 0.30 ^e^	6.53 ± 0.05 ^e^	10.99 ± 0.06 ^d^	0.43 ± 0.03 ^a^	0.18 ± 0.02 ^abc^	88.48 ± 0.04 ^e^
JGR-3	0.65 ± 0.11 ^f^	7.53 ± 0.02 ^b^	14.70 ± 0.18 ^a^	0.17 ± 0.02 ^c^	0.21 ± 0.00 ^a^	89.72 ± 0.01 ^b^

Values are shown by Mean ± SD and values, and different letters within a column indicate significant differences between mean values (*p* < 0.05).

**Table 9:** Molecular structure of glutinous rice starch samples.

Sample	A _(DP 6–12)_	B_1 (DP 13–24)_	B_2 (DP 25–36)_	B_3 (DP≥37)_	ACL	M_w_ (×10^8^ g/mol)
IGR-1	22.43	49.35	13.18	15.05	21.84	1.23
IGR-2	29.82	45.44	12.04	12.71	20.31	1.21
IGR-3	30.68	45.49	13.51	10.32	19.64	1.16
JGR-1	29.90	47.06	11.27	11.77	19.93	1.23
JGR-2	30.73	46.89	12.62	9.76	19.30	1.14
JGR-3	35.35	43.59	9.90	11.16	19.01	1.21

**Table 10:** DSC data of glutinous rice starch samples.

Sample	T_o_ (°C)	T_p_ (°C)	T_c_ (°C)	ΔH (J/g)
IGR-1	76.23 ± 0.11 ^a^	81.35 ± 0.06 ^a^	85.47 ± 0.47 ^a^	13.87 ± 0.51 ^a^
IGR-2	64.13 ± 0.01 ^b^	70.13 ± 0.37 ^b^	75.60 ± 0.51 ^bc^	11.28 ± 1.14 ^b^
IGR-3	64.30 ± 0.10 ^b^	68.81 ± 0.08 ^c^	76.43 ± 0.33 ^b^	9.03 ± 0.65 ^bc^
JGR-1	59.15 ± 3.39 ^c^	66.42 ± 0.16 ^e^	73.31 ± 3.55 ^bcd^	10.89 ± 1.57 ^b^
JGR-2	62.73 ± 0.04 ^b^	67.45 ± 0.21 ^d^	72.30 ± 0.06 ^bcd^	8.10 ± 0.23 ^c^
JGR-3	58.92 ± 0.28 ^c^	64.92 ± 0.41 ^f^	69.80 ± 0.17 ^d^	7.27 ± 1.49 ^c^

Values are shown by Mean ± SD and values, and different letters within a column indicate significant differences between mean values (*p* < 0.05).

## Data Availability

The original contributions presented in the study are included in the article, further inquiries can be directed to the corresponding author.
